# Extracts of Phenolic Compounds from Seeds of Three Wild Grapevines—Comparison of Their Antioxidant Activities and the Content of Phenolic Compounds

**DOI:** 10.3390/ijms13033444

**Published:** 2012-03-13

**Authors:** Stanisław Weidner, Anna Powałka, Magdalena Karamać, Ryszard Amarowicz

**Affiliations:** 1 Department of Biochemistry, University of Warmia and Mazury, Oczapowskiego Street 1A, 10-957 Olsztyn, Poland; E-Mails: weidner@uwm.edu.pl (S.W.) ania.m.wrobel@gmail.com (A.P.); 2 Institute of Animal Reproduction and Food Research of the Polish Academy of Sciences, Tuwima Street 10, 10-747 Olsztyn, Poland; E-Mail: m.karamac@pan.olsztyn.pl

**Keywords:** grapevine seeds, catechins, phenolic acids, redycing power, DPPH radical

## Abstract

Phenolic compounds were extracted from three wild grapevine species: *Vitis californica*, *V. riparia* and *V. amurensis* seeds using 80% methanol or 80% acetone. The total content of phenolic compounds was determined utilizing the Folin-Ciocalteu’s phenol reagent while the content of tannins was assayed with the vanillin and BSA precipitation methods. Additionally, the DPPH free radical scavenging activity and the reduction power of the extracts were measured. The RP-HPLC method was applied to identify the phenolic compounds in the extracts, such as phenolic acids and catechins. The seeds contained large amounts of tannins, catechins and gallic acid and observable quantities of *p*-coumaric acid. The total content of phenolic compounds and tannins was similar in the extracts from *V. californica* and *V. riparia* seeds. However, the total content of total phenolic compounds and tannins in the extracts from *V.* californica and *V. riperia* seeds were about two-fold higher than that in the extracts from *V. amurensis* seeds. Extracts from seeds of the American species (*V. californica* and *V. riparia*) contained similarly high concentrations of tannins, whereas extracts from seeds of *V. amurensis* had approximately half that amount of these compounds. The content of catechin and epicatechin was similar in all extracts. The highest DPPH^•^ anti-radical scavenging activity was observed in the acetonic and methanolic extracts of *V. californica* and *V. riparia* seeds— while the acetonic extract from the *V. californica* seeds was the strongest reducing agent.

## 1. Introduction

The known species of grapevine have been divided into three groups based on their origin: Eurasian, American and East-Asian categories. Grapevine is one of the oldest plant species grown by man. Currently, grapevine plantations cover approximately 10 million ha globally. Grapes are the world’s most important fruit with the annual grape harvest nearing 70 million tons. Each year, the processing of grapes for wine and juice yields approximately 10 million tons of press residues which is composed of 38–52% seeds [1,2].

Grapevine seeds are a rich source of polyphenols, which are characterized by a variety of properties, such as antibacterial and antioxidant activities. Polyphenols also modulate the activity of various enzymatic systems [3,4]. During red wine production, polyphenols are extracted from the grapevine seeds and passed to the wine [5]. There has been increasing interest in the compounds present in grapevine seeds, such as gallic acid, catechin, epicatechin, and a wide variety of procyanidins. Interest in phenolics arose when it was discovered that these compounds were powerful scavengers of free radicals and effective inhibitors of LDL oxidation. Furthermore, phenolics are largely responsible for a dietary anomaly known as the French paradox [6]. This term was coined following certain epidemiological discoveries, such as the low myocardial ischaemia mortality rate despite the elevated consumption of saturated lipids and cholesterol in France [5]. It is thought that the French paradox is a result of the relatively high consumption of red wines by the Mediterranean nations.

Recently, there has been an increase in the number of scientific publications on grapevine phenolic compounds, which is stimulated by a wide-range of beneficial pharmacological effects they produce, and therefore their therapeutic potential. Once the positive effects of phenolics on human health were recognized, extracts from grapevine seeds were introduced as a dietary supplement [6].

Furthermore, grapevine seeds are also a source of edible oil, which is believed to be highly valuable because of its high concentration of unsaturated fatty acids [6]. There is ongoing research regarding the role of grapevine seed extracts as antioxidants in food systems in place of synthetic compounds, which can be harmful to humans [3,7].

The aim of this research is to discover the differences between seeds from three wild grapevine species (*Vitis californica*, *Vitis riparia*, and *Vitis amurensis*) and determine their content of phenolic constituents as well as their antioxidant activities.

## 2. Results and Discussion

The total content of phenolic compounds in extracts was assayed utilizing Folin and Ciocalteu’s phenol reagent (Table 1) and indicates that the total content of phenolic compounds in acetonic extracts was higher than in methanolic extracts. In addition, the extracts from *V. californica* and *V. riparia* seeds displayed a high content of phenolic compounds, 396 and 377 mg/g of acetonic extract and 301 and 308 mg/g of methanolic extract, respectively. A similar trend was observed when the total content of phenolic compounds was analyzed in other calculations (Table 1). The seeds of *V. californica* and *V. riparia* contained high quantities of phenolic compounds; however, the *V. amurensis* seeds contained only low levels of phenolic compounds.

Two methods were used to determine the total content of tannins in acetonic extracts: the vanillin method and the BSA precipitation method. Table 2 shows that all of the analyzed extracts contained condensed tannins. Furthermore, both methods indicated that the extracts from the American vines had similarly high concentrations of tannins. According to the vanillin test, the absorbance of the extracts from *V. californica* and *V. riparia* seeds at 500 nm/mg of extract/mL were 0.142 and 0.130, respectively. Thus our results using the BSA precipitation method were consistent with the vanillin test: the absorbencies at 510 nm/mg of extract/mL were also elevated, reaching 0.059 and 0.051, respectively. Notably, the extracts from *V. amurensis* seeds contained only half the amount of tannins. The contents were determined using the vanillin assay and the BSA precipitation assay and the absorbance at 500 and 510 nm/mg of extract were 0.157 and 0.118, respectively (Table 2).

**Figure 1:**
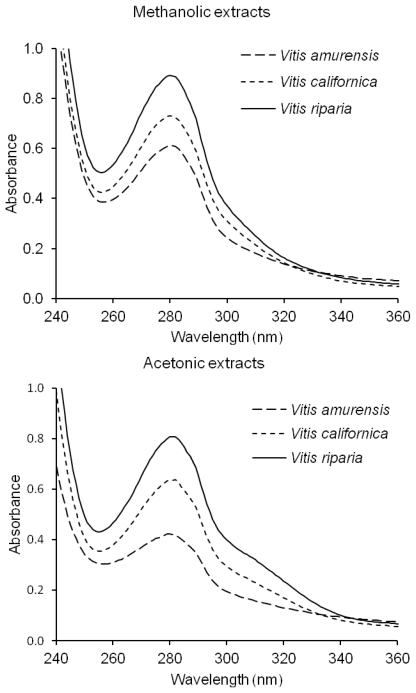
UV spectra of methanolic and acetonic extracts of grapevine seeds.

The spectra of the phenolic compounds derived from the acetonic and methanolic extracts of grapevine seeds have revealed the presence of absorbance maxima at the wavelength of 280 nm, possibly originating from tannins, catechins, and gallic acid derivatives (Figure 1). Additionally, points of inflection were noticed between 295–320 nm in the UV spectra of the phenolic compounds from acetonic extracts perhaps due to the presence of phenolic acids (Figure 1).

**Figure 2:**
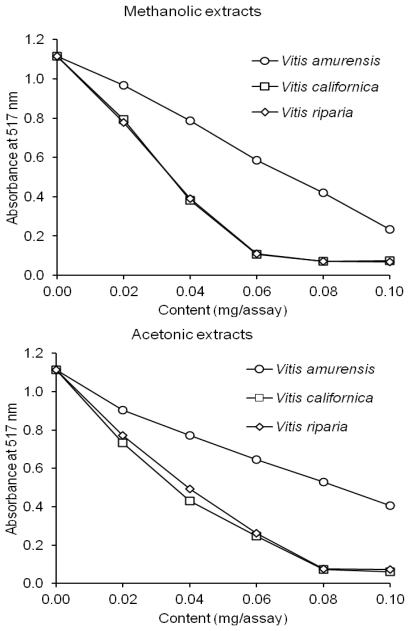
Antiradical activity of methanolic and acetonic extracts of grapevine seeds against 2,2′-diphenyl-1-picrylhydrazyl radical (DPPH) radical.

The DPPH method is based on the spectrophotometric measurement of DPPH radical concentration changes resulting from the DPPH reaction with an antioxidant. During the reaction, DPPH radical with an unpaired valence electron at one atom of nitrogen bridge is reduced by a hydrogen atom from an antioxidant. A freshly prepared DPPH^•^ methanolic solution exhibits a purple color. This purple color generally fades when an antioxidant is present. Thus, antioxidant molecules can quench DPPH^•^, converting it a virtually colorless reaction product (DPPH-H) and decrease the absorbance of the solution. Hence, an increase in the antioxidant content results in a more colorless solution. The results of the analyses are depicted in Figure 2. All the extracts demonstrated a capacity to scavenge the DPPH radical in the test run (Figure 2). Furthermore, the acetonic and methanolic extracts showed a similar antiradical activity (Figure 2). The acetonic and methanolic extracts from the *V. californica* and *V. riparia* seeds demonstrated a higher antiradical of DPPH^•^ scavenging than the extracts from the *V. amurensis* seeds (Figure 2).

**Figure 3:**
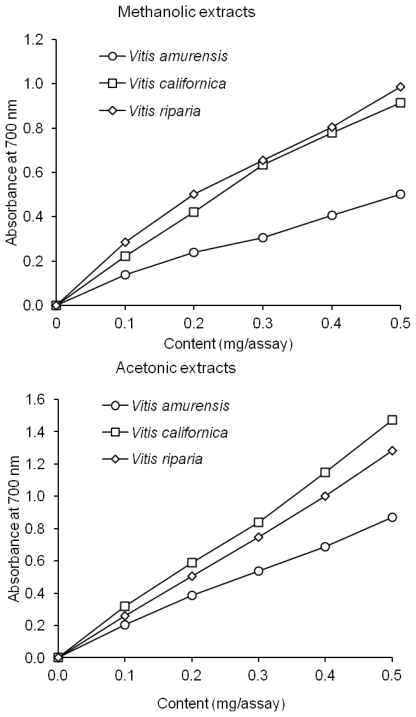
Reducing power of the methanolic and acetonic extracts of grapevine seeds.

The extracts were able to reduce Fe^3+^ ions to Fe^2+^ ions, and the color of the reaction mixture changed from yellow to various shades of blue and green. The amount of the generated Fe^2+^ ions in the solution was measured by analyzing the amount of Prussian blue produced in the solution, using the absorbance measurements at a 700 nm wavelength (Figure 3). The acetonic extracts exhibited a stronger reduction power than the methanolic extracts in all of the assays. The acetonic extract from the *V. californica* seeds was the strongest reducing agent, but the acetonic extract from the *V. riparia* seeds displayed the strongest reducing power. However, both extracts from the American species of grapevine seeds possessed a stronger reducing power than the extracts from *V. amurensis* seeds (Figure 3).

Table 3 shows the catechin content in methanolic extracts and grapevine seeds. Catechin and epicatechin had similar concentrations, ranging between 33 and 38 mg/g of extract. The total content of catechins in these extracts ranged from 66 to 71 mg/g extract. The results presented in Table 3 from other calculations showed similar tendencies, except for a few ones calculated at f.w.

The gallic acid content in methanolic extracts from grapevine seeds is shown in Table 4. The extract from *V. riparia* seeds contained the highest quantity of gallic acid (10.2 mg/g of extract), but the *V. californica* seeds contained significantly less gallic acid in their extracts (8.6 mg/g of extract). It should be emphasized that the gallic acid content in the extracts from *V. amurensis* seeds was several-fold lower (1.6 mg/g of extract) than the extracts from *V. amurensis* and *V. californica* seeds. Significantly more gallic acid was present in the bound form than in the free form in all of the prepared extracts. In the extracts from *V. amurensis* and *V. californica* seeds, the dominant fractions of gallic acid (in equal proportions) were present in theirester- and glycoside-bound forms. In turn, the dominant form of gallic acid in the extracts from *V. riparia* seeds was the ester-bound form. The extract from *V. riparia* seeds contained the highest concentrations (amongst all tested extracts) of free (2.3 mg/g of extract) and ester-bound gallic acid (4.7 mg/g of extract).

This study revealed that the examined grapevine seeds contained large amounts of tannins, catechins, gallic acid and much smaller amount of *p*-coumaric acid (Table 5) only as free and esterified compound (which, however, occurred in a much smaller concentration than gallic acid). These observations correspond with previous studies, which have shown that grapevine seeds are a rich source of condensed tannins (proanthocyanidins) and their monomers (flavan 3-ols or catechins). Condensed tannins and other monomers are mainly located in seed covers and appear to be bound with gallic acid [8–15].

Our data revealed that the extracts obtained from grape seeds of *V. californica* and *V. riparia* were both characterized by a high content of total phenolics and tannins. Conversely, the content of total phenolics and tannins determined in grape seeds of *V. amurensis* was considerably lower. Gallic and *p*-coumaric acid levels in the extracts of *V. californica* and *V. riparia* were also similar, but a much lower content of these phenolic acids was observed in the extract of *V. amurensis*.

The analyses also revealed the presence of two flavan-3-ol isomers, (+)-catechin and (−)-epicatechin, in the extracts from the grapevine seeds. The content of epicatechin and catechin in the analyzed extracts, which is similar to the results reported in earlier studies [16,17]. An increased concentration in plant phenolics may indicate increased protection against various stresses, including oxidative stress [18]. These findings are essential for future studies on the wild grape germplasm, and they may improve the characteristic of grape cultivars. We investigated the antioxidant properties of the extracts from three wild grapevine seeds. It was determined that all of the extracts from the grapevine seeds were characterized by their capacity to scavenge DPPH free radicals and by the reducing power. The antioxidant and antiradical properties of the extracts from the seeds of the American grapevines seeds were much stronger than those of the extracts from the *V. amurensis* seeds. The acetonic extracts from the analyzed grape seeds demonstrated a much stronger antioxidant power than their methanolic counterparts.

We concluded that the antioxidant and anti-radical activities of particular extracts were closely related to the total content of phenolic compounds in those extracts. Thus, our results are consistent with the reported conclusions derived from previous studies [19–21]. It is possible that the high antioxidant capacity of the extracts was attributable to the large content of condensed tannins and catechins. This was confirmed by the extracts from *V. californica* and *V. riparia* seeds, which contain the highest quantities of tannins and display the strongest antioxidant power. Tannins belong to a class of phenolics, whose subunit composition changes in relation to their seed localization [22]. The higher tannin condensation of seeds is responsible for its organoleptic properties. Therefore, the astringency and bitterness demonstrated by these compounds are inversely correlated to their degree of polymerisation [23]. Condensed tannins and their monomers are thought to be very powerful antioxidant substances. It has been shown that they have a high capacity for scavenging free radicals [11,24]. In this respect, catechin dimers and epicatechins are particularly powerful free radical scavengers as their capacity for scavenging free radicals is 18-fold higher than that of ascorbic acid [25]. Da Silva *et al.* [24], who tested the ability of particular components of extracts from *V. vinifera* seeds to scavenge the superoxide anion and hydroxyl free radicals, proved that the compounds belonging to the group of proanthocyanidins and their monomers (catechins) were able to quench these reactive radicals.

Phenolic acids are particularly important antioxidants. Large amounts and smaller quantities of *p*-coumaric acid were detected in the seeds of wild grapevine species. Notably, gallic acid is thought to be an especially powerful antioxidant because of three hydroxy groups in the molecule [26].

Previous findings confirmed that using an aqueous solution of acetone as an extractant improves the extractability of proanthocyanidins and consequently the yield of antioxidant properties rather than any other solvents such as methanol [3,7,10]. In experiments on the antiradical activity carried out by Jayaprakasha *et al.* [7], acetonic extracts from *V. vinifera* seeds were more active in scavenging DPPH free radicals than methanolic extracts. Comparing the results obtained for the extracts from the grapevine seeds, it can be concluded that their reduction power was similar to that of tea extracts [27].

## 3. Experimental Section

### 3.1. Chemicals

All solvents used were of analytical grade unless otherwise specified. Methanol, acetone, hexanes, acetonitrile, potassium ferricyanide, and trichloroacetic acetic were acquired from the P.O.Ch. Company (Gliwice, Poland). Vanillin, Folin & Ciocalteu’s phenol reagent, 2,2′-diphenyl-1-picrylhydrazyl radical (DPPH^•^), (+)-catechin, (−)-epicatechin, gallic acid, and *p*-coumaric acid were obtained from Sigma-Aldrich (Poznań, Poland).

### 3.2. Plant Material

The experiments were conducted on *Vitis amurensis*, *Vitis californica*, *Vitis riparia* seeds supplied by Sandeman Seeds (Lalongue, France). The dry matter (d.m.) of the seeds was determined by drying at 105 °C for 8 h.

### 3.3. Extract Preparation

Seeds were ground in a coffee mill and defatted with hexanes in a Soxhlet apparatus for 6–8 h. Phenolic compounds were then extracted from raw material using 80% (v/v) acetone or methanol at a solids to solvent ratio of 1:10 (w/v), at 50 °C for 30 min [28]. The extraction was carried out in Erlenmeyer flasks using a shaking water bath (Elpan 357, Wrocław, Poland). The extraction was repeated twice more, the supernatans were filtrated and combined, and the organic solvent was evaporated under vacuum at 40 °C in a Büchi rotary evaporator; the remaining aqueous solution was lyophilized.. The prepared extract was stored at −20 °C until analyzed.

### 3.4. Content of Total Phenolics

The content of total phenolics in the crude extracts was estimated using Folin & Ciocalteu’s phenol reagent [29]. (+)-Catechin was used as a standard in this work.

### 3.5. Condensed Tannins

The content of condensed tannins in the crude extract was determined using the modified vanillin assay [30] and BSA precipitation method [31]. Results were expressed as absorbance units at 500 nm/mg of extract/mL and as absorbance units at 510 nm/mg of extract/mL.

### 3.6. UV Spectra

The UV spectra of the extract dissolved in methanol were recorded using a Beckman DU 7500 diode array spectrophotometer (Beckman Coulter, Inc., Brea, CA, USA).

### 3.7. Scavenging of the DPPH Radical

The scavenging effect of phenolics from the extracts was monitored as described by Amarowicz *et al.* [32]. A 0.1 mL methanolic solution containing between 0.02–0.10 mg of extract was mixed with 2 mL of deionized water and then added to a methanolic solution of DPPH^•^ (1 mM, 0.25 mL). The mixture was vortexed for 1 min, left to stand at room temperature for 20 min, and absorbance of the solution was then measured at 517 nm with the spectrophotometer.

### 3.8. Reducing Power

The reducing power of phenolics was determined as described by Oyaizu [33]. A suspension of extract in 1 mL of deionized water was mixed with 2.5 mL of 0.2 M phosphate buffer (pH 6.6) and 2.5 mL of 1% (w/v) potassium ferricyanide. The mixture was incubated at 50 °C for 20 min. Following this, 2.5 mL of 10% (w/v) trichloroacetic acid was added and the mixture was then centrifuged at 1750 g for 10 min. A 2.5-mL aliquot of the upper layer was mixed with 2.5 mL of deionized water and 0.5 mL of 0.1% (w/v) FeCl^3^. The absorbance of the mixture was measured at 700 nm with the spectrophotometer.

### 3.9. HPLC-PAD Analysis of Catechins

Methanolic extract (10 mg) was dissolved in 1 mL of 80% (v/v) methanol and filtered through a 0.45 μm cellulose acetate filter (Millipore) before HPLC analysis. Catechins were analyzed using a Shimadzu HPLC system (Shimadzu Corp., Kyoto, Japan) consisting of two LC-10AD pumps, SCTL 10A system controller and SPD-M 10A photodiode array detector. The chromatography was carried out using a pre-packed LiChrospher 100 RP-18 column (4 × 250 mm, 5 μm; Merck). Elution for 50 min in a gradient system of 5–40% acetonitrile in water adjusted to pH 2.5 with TFA was employed [34]; detector was set at 280 nm, injection volume was 20 μL and the flow rate was 1 mL/min.

### 3.10. HPLC-PAD Analysis of Phenolic Acids

Phenolic acids (free and those liberated from soluble esters and from soluble glycosides) were isolated from the extracts according to the method previously described by Weidner *et al.* [35]. An aqueous suspension of the methanolic extract (200 mg in 20 mL) was adjusted to pH 2 with 6 M HCl, and free phenolic acids were extracted five times into 20 mL of diethyl ether using a separatory funnel. The ether extract was evaporated to dryness under vacuum at room temperature. The water solution was neutralized and then lyophilised. The residue was dissolved in 20 mL of 2 M NaOH and hydrolyzed for 4 h under nitrogen atmosphere at room temperature. After acidification to pH 2 using 6 M HCl, phenolic acids released from soluble esters were extracted from the hydrolysate five times into 30 mL of diethyl ether. Nine milliliters of 6 M HCl were added to the water solution and the solution was placed in nitrogen atmosphere and hydrolyzed for 1 h in a boiling water bath. Phenolic acids released from soluble glycosides were separated from the hydrolysate five times into 45 mL of diethyl ether. After ether evaporation, the dry residue was dissolved in 2 mL of methanol and filtered through a 0.45 μm nylon filter. The sample was injected onto an HPLC column. The same Shimadzu HPLC system was employed. The conditions of the separations were as follows: prepacked LUNA C^18^ column (5 μm, 4.6 × 250 mm; Phenomenex); mobile phase water-acetonitrile-acetic acid (88:10:2, v/v/v) [35]; flow rate of 1 mL/min; injection volume of 20 μL; the detector was set at 280 and 320 nm; oven temperature was 20 °C.

### 3.11. Statistical Analysis

All determinations were performed in this study in triplicate. Results are reported as mean and SD values. Analyses of variance and Tukey’s studentised test were performed at level of *P* < 0.05 to evaluate the significance of differences among mean values.

## 4. Conclusions

The analyzed seeds of grapevines contained large amounts of tannins, catechins and gallic acid as well as observable quantities of *p*-coumaric acid. The total content of phenolic compounds and tannins was similar in extracts from *V. californica* and *V. riparia* seeds and about two-fold higher than that in extracts from *V. amurensis* seeds. Extracts from seeds of the American species (*V. californica* and *V. riparia*) contained similarly high concentrations of tannins, whereas extracts from seeds of *V. amurensis* had about half that amount. On the other hand, the highest antiradical activity of scavenging DPPH^•^ was demonstrated by the acetonic extracts from *V. californica* and *V. riparia* seeds. The acetonic extract from *V. californica* seeds was the strongest reducing agent. The extracts of *V. californica* and *V. riparia* seeds due to high antioxidant activities can be applied for production of nutraceuticals and functional foods.

## Figures and Tables

**Table 1:** Content of total phenolics in the extracts and seeds.

Solvent used for extraction	Species	mg/g extract	mg/g f.m.	mg/g d.m.	μg/seed
80% acetone	*V. amurensis*	208 ± 3 a	12.1 ± 0.2 a	14.0 ± 0.2 a	428 ± 6 a
	*V. california*	396 ± 13 b	28.5 ± 1.0 b	31.7 ± 1.1 b	846 ± 29 b
	*V. riparia*	377 ± 17 b	29.7 ± 1.3 b	32.7 ± 1.5 b	843 ± 38 b

80% methanol	*V. amurensis*	172 ± 3 a	9.2 ± 0.2 a	10.6 ± 0.2 a	324 ± 5 a
	*V. california*	301 ± 6 b	17.5 ± 0.4 b	19.4 ± 0.4 b	518 ± 10 b
	*V. riparia*	308 ± 10 b	19.7 ± 0.7 c	21.7 ± 0.8 c	559 ± 19 b

Means with the same letter are not significantly different (*P* < 0.05). f.m.—fresh matter; d.m.—dry matter.

**Table 2:** Content of condensed tannins in the acetonic extracts expressed as absorbance at 500 and 510 nm.

Species	Vanillin method (Absorbance at 500 nm/mg extract/mL)	BSA precipitation method (Absorbance at 510 nm/mg extract/mL)
*V. amurensis*	0.063 ± 0.001 a	0.024 ± 0.002 a
*V. california*	0.142 ± 0.002 b	0.059 ± 0.002 b
*V riparia*	0.130 ± 0.001 c	0.051 ± 0.001 b

Means with the same letter are not significantly different (*P* < 0.05).

**Table 3:** Content of catechins in the methanolic extracts and seeds.

Species	Phenolic compound	mg/g extract	mg/g f.m.	mg/g d.m.	μg/seed
*V. amurensis*	Catechin	33 ± 2 a	1.7 ± 0.1 a	2.0± 0.1 a	61 ± 3 a
	Epicatecin	35 ± 2 a	1.9 ± 0.1 a	2.2 ± 0.1 a	66 ± 4 a
	Total	68 ± 4 a	3.6 ± 0.2 a	4.2 ± 0.2 a	127 ± 7 a

*V. californica*	Catechin	33 ± 2 a	1.9 ± 0.1 a	2.2 ± 0.1 a	57 ± 3 a
	Epicatecin	38 ± 2 a	2.2 ± 0.1 b	2.4 ± 0.1 a	65 ± 4 a
	Total	71 ± 4 a	4.1 ± 0.2 b	4.6 ± 0.2 a	122 ± 6 a

*V. riparia*	Catechin	31 ± 2 a	2.0 ± 0.1 a	2.2 ± 0.1 a	55 ± 3 a
	Epicatecin	35 ± 2 a	2.2 ± 0.1 b	2.5 ± 0.1 a	63 ± 3 a
	Total	66 ± 4 a	4.2 ± 0.2 bc	4.7 ± 0.2 a	118 ± 6 a

Means for the same phenolic compounds with the same letter are not significantly different (*P* < 0.05); f.m.—fresh matter; d.m.—dry matter.

**Table 4:** Content of gallic acid in the methanolic extracts and seeds.

Species	Form of phenolic acids	mg/g extract	μg/g f.m.	μg/g d.m.	μg/seed
*V. amurensis*	Free	0.3 ± 0.0 a	18 ± 2 a	21± 2 a	0.7 ± 0.1 a
	Esterified	0.7± 0.0 a	34 ± 1 a	40 ± 1 a	1.2 ± 0.0 a
	Glucosided	0.6 ± 0.0 a	33 ± 1 a	38 ± 2 a	1.2 ± 0.1 a
	Total	1.6 ± 0.0 a	85 ± 4 a	99 ± 5 a	3.1 ± 0.1 a

*V. californica*	Free	1.9 ± 0.2 b	112 ±12 b	125 ± 13 b	3.3 ± 0.0 b
	Esterified	3.4 ± 0.1 b	194 ± 4 b	216 ± 5 b	5.8 ± 0.1 b
	Glucosided	3.3 ± 0.2 b	191 ± 10 b	212 ± 11 b	5.7 ± 0.3 b
	Total	8.6 ± 0.5 b	497 ± 26 b	553 ± 29 b	14.8 ± 0.7 b

*V. riparia*	Free	2.3 ± 0.2 b	145 ± 15 b	160 ± 17 c	4.1 ± 0.4 b
	Esterified	4.7 ± 0.1 c	298 ± 6 c	328 ± 7 c	8.5 ± 0.2 c
	Glucosided	3.2 ± 0.2 b	202 ± 11 b	223 ± 12 b	5.7 ± 0.3 b
	Total	10.2 ± 0.5 c	645 ± 32 c	711 ± 36 c	18.3 ± 0.9 c

Means for the same form of phenolic acid with the same letter are not significantly different (*P* < 0.05); f.m.—frsh matter; d.m.—dry matter.

**Table 5:** Content of *p*-coumaric acid in the methanolic extracts and seeds.

Species	Form of phenolic acids	mg/g extract	μg/g f.m.	μg/g d.m.	μg/seed
*V. amurensis*	Free	0.033 ± 0.004 a	1.73 ± 0.22 a	2.00 ± 0.26 a	0.061 ± 0.008 a
	Esterified	0.026 ± 0.002 a	1.38 ± 0.09 a	1.60 ± 0.11 a	0.049 ± 0.003 a
	Total	0.059 ± 0.006 a	3.13 ± 0.31 a	3.62 ± 0.36 a	0.110 ± 0.011 a

*V. californica*	Free	0.026 ± 0.003 b	1.50 ± 0.19 a	1.67 ± 0.21 b	0.045 ± 0.006 b
	Esterified	0.134 ± 0.009 b	7.77 ± 0.54 a	8.64 ± 0.60 b	0.230 ± 0.016 b
	Total	0.160 ± 0.012 b	9.28 ± 0.72 b	10.32 ± 0.71 b	0.275 ± 0.022 b

*V. riparia*	Free	0.016 ± 0.002 c	1.02 ± 0.11 b	1.13 ± 0.00 c	0.029 ± 0.004 c
	Esterified	0.108 ± 0.007 c	6.89 ±0.47 a	7.60 ± 0.90 b	0.196 ± 0.013 c
	Total	0.124 ± 0.010 c	7.91 ± 0.99 b	8.72 ±0.42 c	0.225 ± 0.017 c

Means for the same form of phenolic acid with the same letter are not significantly different (*P* < 0.05).
